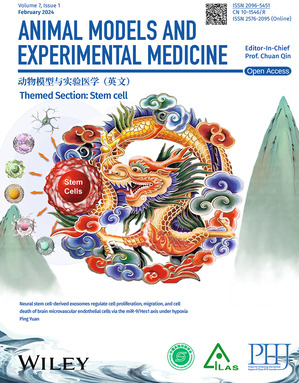# Cover Picture

**DOI:** 10.1002/ame2.12397

**Published:** 2024-03-25

## Abstract

The cover image is based on the article ‘Neural stem cell‐derived exosomes regulate cell proliferation, migration, and cell death of brain microvascular endothelial cells via the miR‐9/Hes1 axis under hypoxia’(DOI:10.1002/ame2.12394) reported by Xiaojun Deng, Xiaoyi Hu, et al. Stem cells, characterized by their undifferentiated nature, possess the remarkable ability for self‐renewal, high proliferation, anti‐inflammation and the potential to differentiate into various cell types. Stems cells, like dragons, symbolize regeneration and hope. These cells play pivotal roles in the body’s growth, development, and the onset of disease, rendering them as highly desirable targets in cell therapy.